# Xuesaitong injection treating acute myocardial infarction: A systematic review and meta-analysis

**DOI:** 10.1097/MD.0000000000027027

**Published:** 2021-09-17

**Authors:** Yingying Hua, Mingjing Shao, Yan Wang, Jinhang Du, Jiaxing Tian, Kangkang Wei, Jiangmeng Chang, Xiaoqiong Zhang, Ming Chen, Jiangquan Liao

**Affiliations:** a Department of Traditional Chinese medicine, Beijing Fuxing Hospital, Capital Medical University, Beijing, China; b National Integrated Traditional and Western Medicine Center for Cardiovascular Disease, China-Japan Friendship Hospital, Beijing, China; c Department of Endocrine, Guang’anmen Hospital, China Academy of Chinese Medical Sciences, Beijing, China; d Graduate School, Beijing University of Chinese Medicine, Beijing, China; e Xiangyang Central Hospital, Affiliated Hospital of Hubei University of Arts and Science. Xiangyang, China; f Department of Cardiology, Jiangmen Wuyi Hospital of Traditional Chinese medicine, Jiangmen, China.

**Keywords:** acute myocardial infarction, meta-analysis, randomized controlled trials, xuesaitong injection

## Abstract

**Background:**

Although the incidence of acute myocardial infarction (AMI) is decreasing, the mortality in AMI patients remains substantial. Traditional Chinese medicine has shown its role in the prevention and management of AMI. The purpose of this study is to evaluate the clinical efficacy of Xuesaitong injection (XST) for the treatment of AMI by a meta-analysis.

**Methods:**

A literature search was performed in 5 medical databases up to June 1, 2020. Randomized controlled trials involving XST combined with conventional treatment versus conventional treatment were included. A meta-analysis of clinical efficacy, left ventricular function and other objective parameters was performed to evaluate the effects of XST on AMI.

**Results:**

Five randomized controlled trials involving 539 participants were eventually included. Meta-analysis showed that the combination of XST and conventional treatment could achieve significantly better effect on improving clinical efficacy (risk ratio: 1.09 [1.01, 1.17]; *P* = .04), left ventricular ejection fraction (mean difference [MD]: 3.18 [1.69, 4.67]; *P* < .0001), hypersensitive C-reactive protein (MD: −2.58 [−5.04, −0.12]; *P* = .04), interleukin 6 (MD: −26.00 [−38.85, −13.16]; *P* < .0001), cardiac troponin T (MD: −15.85 [−18.09, −13.61]; *P* < .00001) and creatine kinase myocardial isoenzyme (MD: −73.06 [−79.74, −66.37]; *P* < .00001).

**Conclusion:**

XST combined with conventional treatment can achieve better efficacy on clinical performance and some of the AMI related parameters. However the interpretation of the results should be cautious, due to the relatively low quality of included trials. More rigorously designed, large-scaled, randomized controlled trials are warranted to support its clinical use in the future.

## 1 Introduction

According to the Global Burden of Death^[1]^ Ischemic Heart Disease (IHD) is the number 1 cause of deaths in cardiovascular diseases both in male and female. IHD has caused 8.9 million deaths in 2017, which has increased 22.3% from 2007^[4]^. Although the circumstance of IHD is far from satisfying, the incidence rate of ST-segment elevation myocardial infarction (STEMI) is decreasing around the world.^[2,3]^ It can be attributed to the greater use of reperfusion therapy, primary percutaneous coronary intervention (PCI), modern antithrombotic therapy, and secondary prevention.^[4,5]^ Yet there are still some challenges to overcome, such as angina pectoris after PCI, higher requirement of the physicians and equipment, relatively more cost for new surgical and medication therapy. The evidence for some of the newly invented antiplatelet, anticoagulant and lipid-lowering therapy is still insufficient. Traditional Chinese medicine (TCM) has shown its role in the prevention and management of IHD. Numerous researches were held within the framework of modern medicine, some of which have proven the efficacy of TCM.^[6–8]^

Xuesaitong injection (XST) is one of the major TCM patent medicine used in IHD. Its major components are saponins from *Panax notoginseng* (PNS), including gin-senoside Rb1, ginsenoside Rg1, and notoginsenoside R1.^[9]^ Researches have indicated that XST can inhibit platelet aggregation, increase blood flow, improve left ventricular (LV) diastolic function in hypertensive patients, and has anti-inflammatory effect.^[10,11]^ The clinical application of XST in acute myocardial infarction (AMI) is widely accepted in China, and some randomized controlled trials (RCTs) have been conducted. However the evidence of XST treating AMI has not been systematically reviewed. In this research, we evaluated the effect of XST through a rigorous systematic review and meta-analysis of randomized trials.

## 2 Methods

We conducted this meta-analysis in accordance with the Preferred Reporting Items for Systematic Reviews and Meta-Analyses guidelines^[12]^

### 2.1 Search strategy

Two authors searched for potential articles independently through Cochrane Library, Medline, Embase, China National Knowledge Infrastructure and Wanfang database, from their inception to June 1, 2020. The following medical subject headings and free terms adapted to each database were used in the literature search: (AMI or myocardial infarction or STEMI) and (XST or Xuesaitong or Xue sai tong). No language restriction was applied. The authors of the articles were contacted for detailed information if necessary.

### 2.2 Study selection

Two authors independently screen and judged the eligible researches according to the following criteria: study participants were diagnosed as AMI, ready for revascularization, including PCI, coronary artery bypass grafting or thrombolysis; study should be randomized clinical trial, which compared the efficacy or quantitative parameters of XST with placebo or contemporary medication; and follow-up in each study should ≥2 weeks. For discrepancies in the process of selection, whether to include or exclude a study was resolved by consensus with other investigator.

### 2.3 Data extraction and quality assessment

Two authors independently extracted the following data from the included researches: the name of first author, year of publication, sample sizes of each groups, sex and age of patients, types and duration of interventions, and outcomes. The methodological quality of included trials was assessed using the Cochrane Handbook for Systematic Reviews of Intervention: sequence generation (selection bias), allocation concealment (selection bias), blinding of patients and personnel (performance bias), blinding of outcome assessors (detection bias), incomplete outcome data (attrition bias), selective reporting (reporting bias), and other biases. Each of them was categorized as 3 levels: “low risk”, “unclear risk” and “high risk”, according to the Cochrane Handbook. Any conflicts during this process were solved by discussion or consultation with a third reviewer.

Ethical approval was not necessary due to the data we used in this study was extracted from public database.

### 2.4 Statistical analysis

Statistical analyses were performed via Review Manage software (version 5.3) from the Cochrane Collaboration. Dichotomous data was summarized as risk ratio (RR) with its corresponding 95% confidence interval. Continuous data was summarized as mean difference (MD) with its corresponding 95% confidence interval. Heterogeneity between trials was assessed using I^2^ statistics. If heterogeneity is considered high (50% < I^2^), random effects model would be applied; otherwise heterogeneity is considered low (I^2^ ≤ 50%), a fixed-effect model would be used.^[13]^

## 3 Results

### 3.1 Literature search and study characteristics

**Figure 1:**
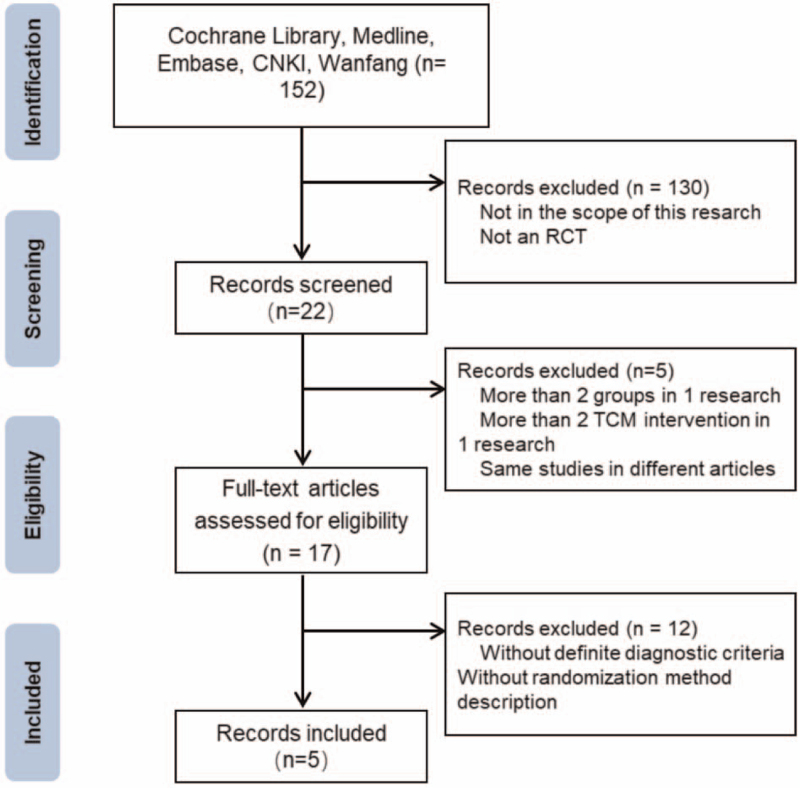
Flow diagram of the RCT inclusion process. RCT = randomized controlled trial, TCM = traditional Chinese medicine.

A flow chart showed the searching and screening process (Fig. 1). Eventually 5 RCTs^[14–18]^ with 539 participants were included in this systematic review. All RCTs were published in Chinese from 2016 to 2019. The detailed information of the included researches were listed in Table 1. Since the included researches were focused on AMI, the duration of treatment were 2 weeks in all of the 5 RCTs, none of them had relatively long term follow-up. The participants in control group (CG) were treated with PCI, antiplatelet and lipid-lowering therapy. Participants in experiment group (EG) were injected with XST before or after PCI, 200 mg per day for 2 weeks along with conventional treatment in CG. Clinical efficacy, LV function, inflammation, myocardial injury related indexes and adverse effects were measured between EG and CG.

**Table 1:** The detailed information of the included researches.

ID	First author	Publication year	Sample size (EG/CG)	Sex (M/F)	Age (yr)	Intervention of EG	Intervention of CG	Course (d)	Outcome indexes
Qiao 2016	Zhili Qiao	2016	40/40	46/34	EG: 62.1 ± 7.9; CG: 63.5 ± 7.8	XST injection + WM	WM	14	hs-CRP, BNP
Xin 2018	Danzhen Xin	2018	54/53	64/43	EG: 51.9 ± 8.4; CG: 52.3 ± 8.2	XST injection + WM	WM	14	ECG, LVEF, BNP, hs-CRP, TNF-α
Zhou 2018	Shu Zhou	2018	62/62	68/56	EG: 55.35 ± 7.24; CG: 54.79 ± 7.45	XST injection + WM	WM	14	LVEF, BNP, cTnT, CK-MB, TNF-α, IL-6, hs-CRP
Chen 2019	Zhaodong Chen	2019	54/54	63/45	EG: 59.87 ± 4.31; CG: 59.79 ± 4.28	XST injection + WM	WM	14	cTnT, CKMB, BNP, LVEF, LVEDD
Zhang 2019	Zhigang Zhang	2019	60/60	79/41	EG: 51.13 ± 8.98; CG: 50.87 ± 7.94	XST injection + WM	WM	14	LVEF, BNP, hs-CRP, TNF-α

BNP = B-type natriuretic peptide, CG = control group, CK-MB = creatine kinase myocardial isoenzyme, cTnT = cardiac troponin T, EG = experiment group, hs-CRP = hypersensitive C-reactive protein, LVEF = left ventricular ejection fraction, TNF-α = tumor necrosis factor α, XST = Xuesaitong injection.

### 3.2 Risk of bias assessment

**Figure 2:**
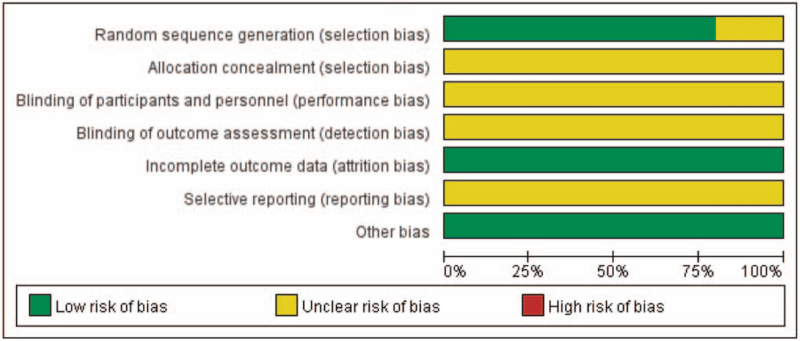
Risk of bias graph.

All of the included studies were single-centered, open-label, parallel designed RCTs. Four of them had described the method of random sequence generation. None of the trials describe allocation concealment. None of the trials had a pretrial estimation of sample size, which indicated the lack of statistical power to ensure appropriate estimation of the therapeutic effect (Fig. 2).

### 3.3 Clinical efficacy

Clinical efficacy was defined as the amelioration of AMI related clinical syndromes, such as angina pectoris, suffocation, and stamina decline. According to the included researches, clinical syndromes relieve more than 75% after intervention is considered ‘effective’ and documented. This criteria is based on <The Guiding Principle of Clinical Research on New Traditional Chinese Drugs>.^[19]^

**Figure 3:**
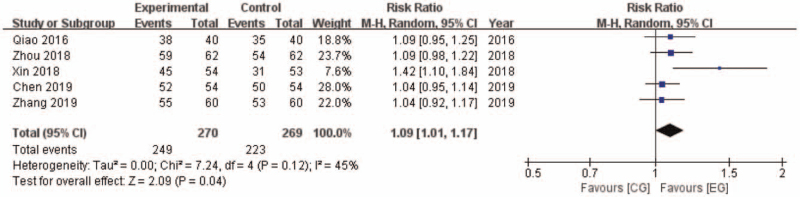
Clinical efficacy comparison between EG and CG. CG = control group, CI = confidence interval, EG = experiment group.

Five researches reported clinical efficacy between EG and CG. 249 of 270 participants (92.22%) achieved effective in EG, while 223 of 269 participants (82.90%) achieved effective in CG. The outcome shows a statistically significant difference in favor of EG (RR: 1.09 [1.01, 1.17]; *P* = .04) (Fig. 3). It is suggested that XST plus conventional treatment had a better effect on relieving symptoms of AMI after PCI.

### 3.4 Left ventricular function

**Figure 4:**
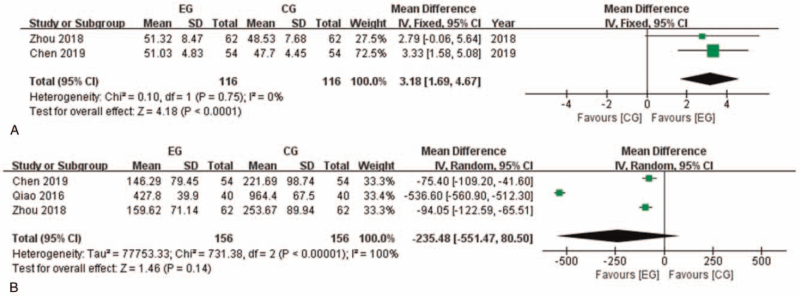
Left ventricular function related indexes comparison between EG and CG. A: LVEF comparison between EG and CG. B: BNP comparison between EG and CG. BNP = B-type natriuretic peptide, CG = control group, CI = confidence interval, EG = experiment group, LVEF = left ventricular ejection fraction.

Two of the researches with 232 participants reported left ventricular ejection fraction (LVEF) after intervention (Fig. 4). Heterogeneity in this analysis is considered low (I^2^ = 0%), thus Fixed model was applied. Meta-analysis showed that LVEF in EG is significantly higher than in CG (MD: 3.18 [1.69, 4.67]; *P* < .0001). Three of the researches with 312 participants reported B-type natriuretic peptide (BNP) after intervention. BNP in EG tended to be lower than CG but with no significance (MD: −235.48 [−551.47, 80.50]; *P* = .14). It is worth to mention that BNP level in 1 of the researches is much higher than the other 2 researches. After removing this research from meta-analysis, heterogeneity was considered low (I^2^ = 0%). The pooled analysis showed significant difference favoring EG (MD: -86.29 [−108.10, −64.48]; *P* < .00001). It indicated that the meta-analysis result of BNP comparison between groups should be interpreted with caution.

### 3.5 Inflammation

**Figure 5:**
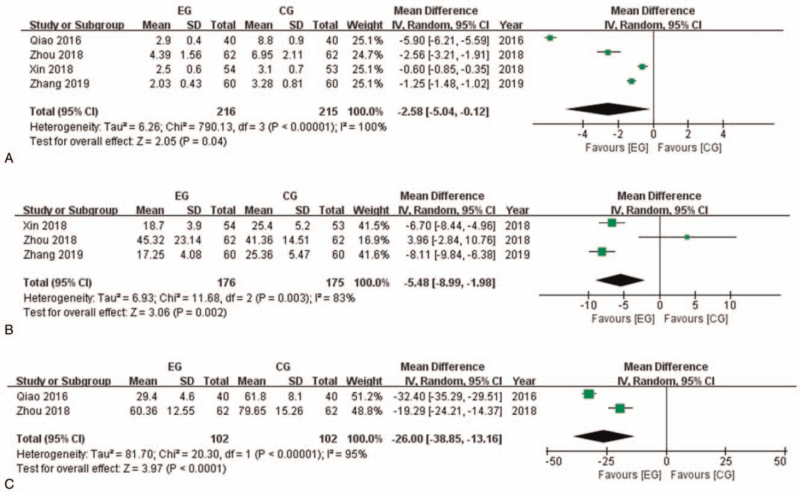
Inflammation related indexes comparison between EG and CG. A: hs-CRP comparison between EG and CG. B: TNF-α comparison between EG and CG. C: IL-6 comparison between EG and CG. CG = control group, CI = confidence interval, EG = experiment group, hs-CRP = hypersensitive C-reactive protein, IL-6 = interleukin 6, TNF-α = tumor necrosis factor α.

Most of the included researches documented inflammation related laboratory indexes. Four of the researches with 431 participants reported hypersensitive C-reactive protein (hs-CRP) after intervention (Fig. 5). Meta-analysis showed that hs-CRP in EG is significantly lower than in CG (MD: −2.58 [−5.04, −0.12]; *P* = .04). Three of the researches with 351 participants reported tumor necrosis factor α (TNF-α) after intervention. Meta-analysis showed that TNF-α in EG is significantly lower than in CG (MD: −5.48 [−8.99, −1.98]; *P* = .002). Two of the researches with 204 participants reported interleukin 6 (IL-6) after intervention. Meta-analysis showed that IL-6 in EG is significantly lower than in CG (MD: −26.00 [−38.85, −13.16]; *P* < .0001).

### 3.6 Myocardial injury

**Figure 6:**
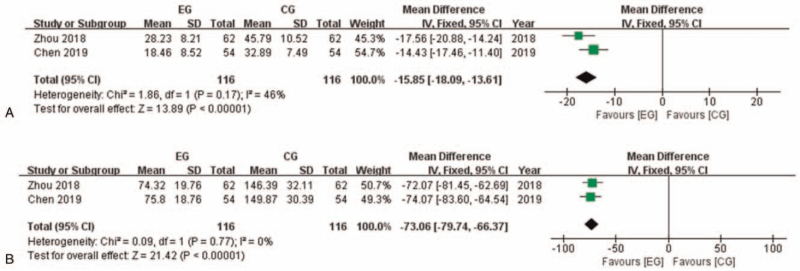
Myocardial injury related indexes comparison between EG and CG. A: cTnT comparison between EG and CG. B: CK-MB comparison between EG and CG. CG = control group, CI = confidence interval, EG = experiment group, cTnT = cardiac troponin T, CK-MB = creatine kinase myocardial isoenzyme.

Two of the researches with 232 participants reported cardiac troponin T (cTnT) after intervention (Fig. 6). Heterogeneity in this analysis is considered low (I^2^ = 46%), thus Fixed model was applied. Meta-analysis showed that cTnT in EG is significantly lower than in CG (MD: −15.85 [−18.09, −13.61]; *P* < .00001). The same researches reported creatine kinase myocardial isoenzyme (CK-MB) after intervention. Fixed model was applied (I^2^ = 0%) and meta-analysis showed that CK-MB in EG is significantly lower than in CG (MD: −73.06 [−79.74, −66.37]; *P* < .00001).

### 3.7 Adverse events

**Figure 7:**
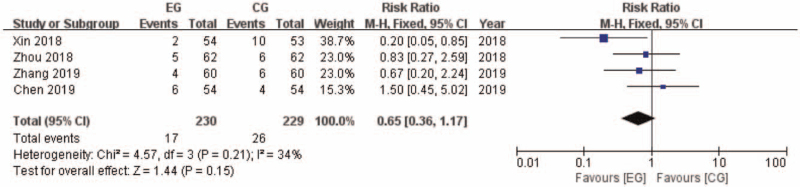
AE comparison between EG and CG. AE = adverse events, CG = control group, CI = confidence interval, EG = experiment group.

Four of the included researches with 459 participants documented adverse events (AE) after intervention (Fig. 7). There were 17 AE in 230 participants in EG, while 26 AE in 229 participants in CG. Heterogeneity in this analysis is considered low (I^2^ = 34%), thus fixed model was applied. The RR was 0.65 favoring EG ([0.36, 1.17]; *P* = .15; I^2^ = 34%).

## 4 Discussion

Although the incidence of STEMI is decreasing, the mortality in STEMI patients remains substantial. The acute and long-term mortality of STEMI are influenced by many factors, more timely use of reperfusion therapy, PCI and modern antithrombotic therapy are beneficial to the patients.^[20,21]^ After vascular recanalization, there are still some clinical issue remain to be solved, such as ischeamic/reperfusion injury, alleviation of angina pectoris, complication of AMI. With the adjunctive use of TCM, better clinical performance and outcomes can be achieved.^[22]^ XST is one of the major TCM patent medicine used in IHD. In this meta-analysis, we have pooled 5 RCTs with 539 participants and have shown that XST combined with western medicine is effective for the treatment of AMI after vascular recanalization. XST not only improves the total clinical efficacy, but also improves LV function, ameliorates inflammation and myocardial injury in the acute and sub-acute stage of STEMI. The AE were considered no significant difference in XST and control group.

XST is one of the most frequently used Chinese patent medicine in clinical practice. It is commonly administered to promote blood circulation and remove blood stasis, hence it is widely used in coronary and cerebral artery diseases. In present, 3 meta-analysis published concerning XST for the treatment of stroke (acute, post, and recovery), and 1 meta-analysis for unstable angina. Our study focused on the very edge of AMI. Persistence or recurrence of angina after successful PCI represent an important clinical issue involving about one third of patients undergoing myocardial revascularization.^[22,23]^ In our study, all of the participants underwent revascularization, and meta-analysis showed that XST can provide complementary and effective treatment for STEMI.

There were still insufficiencies in this study. First, all of the included studies were conducted in China and published in Chinese, which meant that the effects on other races are uncertain. Second, all of the included studies were single-centered, and the scale was relatively small. Third, allocation and blinding methods were not mentioned in all of the studies. These limitations lead to a cautious interpretation of the results. Despite these limitations, our study provided a comprehensive evaluation of the efficacy and safety of XST for the treatment of STEMI.

## 5 Conclusions

In summary, this study has provided evidence that XST combined with conventional treatment can achieve better efficacy on clinical performance and LVEF improvement, hs-CRP, TNF-α, IL-6, cTnT, and CK-MB lowering. The interpretation of the results should be cautious, due to the relatively low quality of included trials. More rigorously designed, large-scaled, randomized controlled trials are warranted to support its clinical use in the future.

## Author contributions

YH and JL designed and supervised the study. YH, MS, and JD carried out the search and screening criteria. JT, KW, JC, XZ, and MC performed literature search, screening, and data collection. YH, MS, and YW drafted the manuscript and JD, JL revised it.

**Conceptualization:** Yingying Hua, Jiangquan Liao.

**Data curation:** Yingying Hua, Kangkang Wei.

**Formal analysis:** Yingying Hua, Mingjing Shao.

**Funding acquisition:** Jiangquan Liao.

**Investigation:** Mingjing Shao, Jinhang Du, Kangkang Wei.

**Methodology:** Jinhang Du, Kangkang Wei, Jiangmeng Chang.

**Project administration:** Xiaoqiong Zhang.

**Software:** Yan Wang, Jiaxing Tian, Xiaoqiong Zhang.

**Supervision:** Jinhang Du, Jiangquan Liao.

**Validation:** Jiaxing Tian.

**Visualization:** Jiaxing Tian.

**Writing – original draft:** Yan Wang, Jiangmeng Chang, Xiaoqiong Zhang, Ming Chen.

**Writing – review & editing:** Yingying Hua.
